# The ROS-induced cytotoxicity of ascorbate is attenuated by hypoxia and HIF-1alpha in the NCI60 cancer cell lines

**DOI:** 10.1111/jcmm.12207

**Published:** 2013-12-14

**Authors:** Tobias Sinnberg, Seema Noor, Sascha Venturelli, Alexander Berger, Paul Schuler, Claus Garbe, Christian Busch

**Affiliations:** a Division of Dermatologic Oncology, Department of Dermatology and Allergology, University of TuebingenTuebingen, Germany; b Department of Internal Medicine I, Medical University HospitalTuebingen, Germany; c Institute of Organic Chemistry, University of TuebingenTuebingen, Germany

**Keywords:** ascorbate, cancer, GLUT-1, HIF-1α, hypoxia, ROS, therapy

## Abstract

Intravenous application of high-dose ascorbate is used in complementary palliative medicine to treat cancer patients. Pharmacological doses of ascorbate in the mM range induce cytotoxicity in cancer cells mediated by reactive oxygen species (ROS), namely hydrogen peroxide and ascorbyl radicals. However, little is known about intrinsic or extrinsic factors modulating this ascorbate-mediated cytotoxicity. Under normoxia and hypoxia, ascorbate IC_50_ values were determined on the NCI60 cancer cells. The cell cycle, the influence of cobalt chloride-induced hypoxia-inducible factor-1α (HIF-1α) and the glucose transporter 1 (GLUT-1) expression (a pro-survival HIF-1α-downstream-target) were analysed after ascorbate exposure under normoxic and hypoxic conditions. The amount of ascorbyl radicals increased with rising serum concentrations. Hypoxia (0.1% O_2_) globally increased the IC_50_ of ascorbate in the 60 cancer cell lines from 4.5 ± 3.6 mM to 10.1 ± 5.9 mM (2.2-fold increase, *P* < 0.001, Mann–Whitney *t*-test), thus inducing cellular resistance towards ascorbate. This ascorbate resistance depended on HIF-1α-signalling, but did not correlate with cell line-specific expression of the ascorbate transporter GLUT-1. However, under normoxic and hypoxic conditions, ascorbate treatment at the individual IC_50_ reduced the expression of GLUT-1 in the cancer cells. Our data show a ROS-induced, HIF-1α-and O_2_-dependent cytotoxicity of ascorbate on 60 different cancer cells. This suggests that for clinical application, cancer patients should additionally be oxygenized to increase the cytotoxic efficacy of ascorbate.

## Introduction

Despite its controversial history in cancer therapy in the 1970s and 1980s [1,2], a large body of evidence has emerged in recent years supporting the hypothesis that high-dose ascorbic acid (AA, ascorbate, vitamin C) kills cancer cells *in vitro* and *in vivo* [3–6].

This cytotoxic effect is driven by the extra-cellular production of reactive oxygen species (ROS), *e.g*. hydrogen peroxide [7,8], making high-dose ascorbate a pro-oxidative anticancer pro-drug. Present limitations to transfer this therapeutic concept to the clinics effectively are the high tissue concentrations needed to elicit a ROS-concentration above a toxic threshold. In this context, we recently demonstrated that patients afflicted with metastatic melanoma (clinical stage IV) have significantly lower plasma ascorbate levels compared with healthy controls and that polychemotherapy or immunotherapy further decreases plasma ascorbate levels in stage IV melanoma patients, and concluded that ascorbate substitution in physiological doses could be considered for late-stage melanoma patients [9]. In humans, the ascorbate concentration required to induce cytotoxicity in cancer cells can only be achieved *via* intravenous administration [9]. Ten-to 30 mM ascorbate serum peak concentrations are achievable through the i.v. administration of 25–100 g ascorbate [4]. In experimental tumour xenografts in mice, the intra peritoneal administration of 4 g ascorbate per kilogram of bw results in the accumulation of 20–40 mM ascorbate and 500 nM ascorbate radical in the tumours [4]; however, it remains to be determined to which molarity ascorbate can be accumulated in malignant tumours and their metastases in human cancer patients. A large number of studies have observed an increased generation of ROS and the alteration of the redox status in cancer cells, which are more vulnerable to the increased oxidative stress induced by exogenous ROS-generating compounds that inhibit the endogenous antioxidant system [10]. In line, it was speculated that an exogenous increase in ROS stress in cancer cells might cause an elevation of ROS above a toxic threshold (thus overwhelming the antioxidant capacity of the cell), which possibly provides a biochemical basis to apply therapeutic strategies to selectively kill cancer cells by using ROS-mediated mechanisms [11].

In addition to having an altered ROS status, cancer cells and the tumour microenvironment are frequently hypoxic [12]. Hypoxia is a critical hallmark of solid tumours and involves enhanced cell survival, angiogenesis, glycolytic metabolism and metastasis [13–15]. Interestingly, a recent review on hyperbaric oxygen (HBO) treatment concluded that HBO can be inhibitory and reduce cancer growth in some cancer types [16].

In endometrial cancer, low ascorbate levels are associated with high hypoxia-inducible factor-1α (HIF-1α) activation, HIF-1α-mediated up-regulation of glucose transporter 1 (GLUT-1) and an aggressive tumour phenotype [17]. Most cells maintain ascorbate concentrations at low millimolar levels either by active transport *via* sodium-dependent vitamin C transporter (SVCT) that transport the reduced form of ascorbic acid or *via* the hexose transporter, GLUT-1, which transports dehydroascorbic acid (the oxidized form of ascorbic acid) competitively with glucose [18]. Thus, the level of GLUT-1-expression might be an indicator for the susceptibility of cancer cells towards ascorbate-induced cell death.

Regarding the currently observed limited efficacy of high-dose ascorbate on the killing of tumour cells in cancer patients in clinical phase I trials [10,19], in the present study, we analysed whether hypoxic conditions might, in part, be responsible for cancer cell resistance towards ascorbate-induced cell death. In detail, we investigated whether (*i*) the ascorbate-induced generation of ROS was serum-dependent, (*ii*) the susceptibility to ascorbate-induced cytotoxicity in the 60 cancer cell lines of the NCI60 panel was merely influenced by exogenous O_2_ availability and/or by HIF1α and (*iii*) correlated with the endogenous GLUT-1 expression. The cell lines of the NCI60 panel were chosen for this study as (*i*) they are well-defined and well-characterized (mutation status, RNA expression, *etc*.), (*ii*) they are the standard tool used for cytotoxicity testing in the screening of novel drugs by the NCI and (*iii*) as they consist of different tumour entities, thus enabling a detailed comparison.

Our results indicate that the generation of ROS from ascorbate is catalysed by serum and that severe hypoxia (as present in cancer metastases) mediates a reduced susceptibility (or resistance) of all 60 cancer cell lines towards ascorbate-induced inhibition of cell proliferation (driven by induction of apoptosis). This ascorbate resistance was enhanced by cobalt chloride (CoCl_2_)-induced HIF1α [20]. However, the ascorbate-induced cytotoxicity did not correlate with the endogenous GLUT-1 expression. These data suggest that a clinical application of high-dose ascorbate on cancer patients should be complemented by oxygenation (*e.g. via* HBO treatment) of the patient to assure for adequate tissue oxygen levels necessary for ascorbate-driven ROS generation. This might improve the therapeutic anti-cancer properties of high-dose ascorbate in the cancer patient.

## Materials and methods

### Electron spin resonance spectroscopy (ESR)

To detect the induction of ascorbyl radicals by ascorbate, ESR was applied. Ascorbyl radical induction was measured in medium with 8 mM ascorbate. Electron spin resonance spectroscopy ascorbyl spectra were recorded as described previously [21]. Briefly, the samples were measured in a quartz flat cell (60 × 17 × 0.7 mm^3^) on a Bruker ESP300E x-band spectrometer (Rheinstetten, Germany) equipped with a TM 4103 resonator operating at 9.8 GHz. Instrument settings were as follows: modulation frequency 100 kHz; microwave power 5 mW; modulation amplitude, gain and time constant varied within the range of ascorbyl radicals. Electron spin resonance spectroscopy measurements were performed in triplicates.

### Measurement of intracellular peroxide radicals (H_2_O_2_)

For the generation of peroxide radicals (H_2_O_2_), nine different cell lines were exposed to 8 or 16 mM ascorbate or to 0.5 mM H_2_O_2_, with or without the addition of 100 μg/ml catalase in RPMI full medium (refer to Cells and cell culture for details) for 1 hr. The medium was discarded, cells were washed once with PBS and H_2_O_2_ was measured in PBS by using dichlorofluorescein substrate (Invitrogen, Darmstadt, Germany) according to the procedure described by the supplier. Experiments were performed in quadruplicates.

### Cells and cell culture

All 60 cell lines (NCI60 cells, Table 1, purchased from the National Cancer Institute, Bethesda, MD, USA), were cultured in RPMI 1640 medium supplemented with 10% foetal bovine serum (FBS), 1% penicillin and streptomycin and 1% L-glutamine. The medium was changed at 48 hrs intervals, and the cells were passaged upon 90% confluence. All cell cultures were maintained at 37°C in a 95% air/5% CO_2_ atmosphere at 100% humidity. For hypoxia experiments, the normoxic O_2_ level (21%) was reduced to 0.1% O_2_ (hypoxia-incubator used: Galaxy 48R, New Brunswick [Eppendorf], Hamburg, Germany).

**Table 1:** IC_50_ values of ascorbate under normoxia (21% O_2_) and hypoxia (0.1% O_2_)

	IC_50_ ascorbate (21% O_2_)	IC_50_ ascorbate (0.1% O_2_)	Fold increase
Renal	5.2	12.8	2.5
Breast	2.4	6.8	2.8
Prostate	13.2	22.9	1.7
Colon	3.7	8.6	2.3
Lung	5.9	12.2	2.1
Leukaemia	0.6	1.2	2.0
Melanoma	3.1	9.7	3.1
Ovarian	3.7	7.9	2.1
Glioblastoma	3.0	8.8	2.9
Mean	4.5	10.1	2.2
SD	3.6	5.9	

### Ascorbate and CoCl_2_ treatment

The following chemicals were used: Injectable vitamin C solution (Pascorbin®, 150 mg ascorbate/1 ml injection solution, pH7.0; Pascoe pharmazeutische Praeparate GmbH, Giessen, Germany), CoCl_2_ and catalase (both from Sigma-Aldrich, Hamburg, Germany). Ascorbate or CoCl_2_ was added directly to the culture medium of the cells. Cells treated with culture medium only served as controls. 100 μM CoCl_2_ was applied for 24 hrs before ascorbate treatment or harvesting the cells to induce HIF1α.

### Proliferation assay

Cells were seeded as triplicates in 96-well plates at a density of 2500 cells per well in 50 μl medium. After 24 hrs, medium was replaced by medium containing ascorbate in rising concentrations to determine the IC_50_ concentration for each cell line. The following concentrations of ascorbate were applied to determine the IC_50_ value: 32, 16, 8, 4, 2 and 1 mM, 500, 250, 125, 62.5, and 31.25 μM. Cells exposed to culture medium or culture medium only served as controls. All experiments were performed in quadruplicates. The exact IC_50_ value was calculated by using GraphPad Prism 5 (Nonlinear Regression EC_50_ shift). The assay was started following incubation for 24 hrs. Medium was discarded, each well was washed twice with PBS (without Ca^2+^ and Mg^2+^) and 100 μl of a solution containing 100 mg 4-methylumbelliferyl heptanoate per ml PBS was added. Plates were incubated at 37°C for 1 hr and measured in a Fluoroskan II (Labsystems, Helsinki, Finland) with an λ_em_ of 355 nm and an λ_ex_ of 460 nm. The intensity of fluorescence indicates the number of viable cells in the wells [22].

### FACS cell-cycle analysis

Cells were treated with ascorbate at their respective IC_50_ concentrations (as determined by the proliferation assay described above). After 24 hrs, cells (1 × 10^6^) were harvested, washed with cold PBS, fixed with 70% ethanol and incubated at 4°C over night. Cells were then centrifuged and washed twice in cold PBS. Intracellular DNA was labelled with propidium iodide solution [propidium iodide 40 mg/ml (Sigma-Aldrich) and RNase 100 mg/ml (Thermo Scientific, Dreieich, Germany) in PBS] and incubated at 4°C for 30 min. in the dark. Cell cycle was analysed by using flow cytometry and FACSDiva software (BD Biosciences, Heidelberg, Germany). 100,000 cells were analysed per treatment group.

### Immunoblotting

Cells were seeded into T75 flasks and incubated for 24 hrs. Medium was replaced by medium containing ascorbate at IC_50_ concentrations, and cells were further incubated for 24 hrs. Cells were next washed with PBS and lysed for 20 min. on ice with a lysis buffer (10 mM Tris-HCl pH7.4, 200 mM NaCl, 1 mM EDTA, 10% Triton). Cell lysates were cleared by centrifugation for 5 min. at 13.000 g at 4°C and 20 μg protein subjected to SDS–PAGE and transferred to polyvinylidene difluoride membranes. After blocking in PBS/0.1% Tween-20/5% dry milk for 1 hr at RT, the blots were incubated overnight with the primary antibodies directed against anti-GLUT-1 (1:1000; Abcam, Cambridge, United Kingdom), anti-HIF1α (0.5 μg/ml; R&D Systems, Wiesbaden, Germany) and against anti-β-actin (1:000; Cell Signaling, Frankfurt, Germany), in PBS/0.1% Tween-20/5% dry milk, washed with PBS for 3 × 10 min. and incubated with secondary HRP-conjugated antibody (Cell Signaling, Frankfurt, Germany). After washing with PBS for 3 × 10 min., the ECLplus detection reagent (GE Healthcare, Munich, Germany) was used for detection of. The membrane was immersed in ECL solution for 1 min. and then exposed to X-ray film (Eastman Kodak, Rochester, NY, USA). For the detection of HIF1α, a CCD-camera was used (BioChemi, Ultra-Violet Products Ltd, Cambridge, United Kingdom). For densitometric analysis of the Western blot bands, we used ImageJ software or LabWorks analysis software (Ultra-Violet Products Ltd). GLUT-1 and HIF1α protein bands were normalized to the respective actin band.

### Statistical analysis

Statistical analyses were performed with a two-tailed unpaired *t*-test and Mann–Whitney *t*-test. *P* < 0.05 was considered statistically significant. Fitted midpoints of the two dose–response curves (logEC_50_) under normoxia and hypoxia were statistically compared by using the *F*-test function for non-linear curve fits of GraphPad Prism 5 and *P* < 0.05 was considered significant. Two-way anova was used for the statistical multiple comparisons of ascorbate-treated cells in combination with hypoxia and/or CoCl_2_ treatment.

## Results

### Ascorbate generates ascorbyl and intracellular peroxide radicals in medium and in nine different cancer cell lines

**Figure 1:**
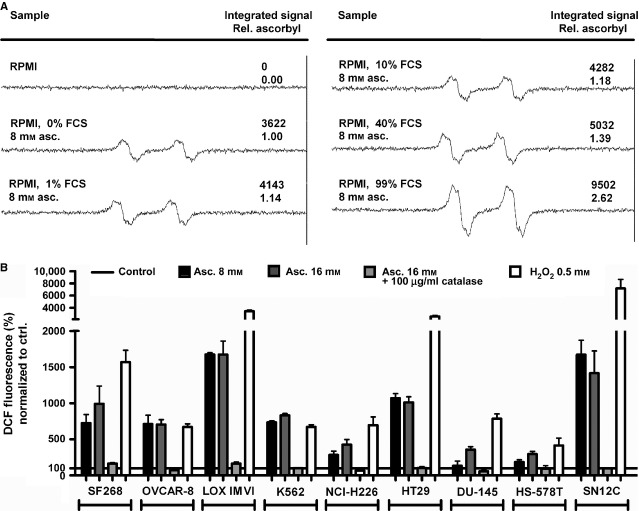
Ascorbate generates ascorbyl and intracellular peroxide radicals in medium and in nine different cancer cell lines. (A) Induction of Asc^−•^ radicals from ascorbate in medium, measured by electron spin resonance (ESR) spectroscopy. Increasing serum concentrations yield more detectable Asc^−•^ radicals. All ESR measurements were performed in triplicates; shown is one representative image for each treatment group. (B) Nine different cancer cell lines were exposed to ascorbate (8 or 16 mM) with and without the addition of 100 μg/ml catalase in full medium. The generation of H_2_O_2_ after ascorbate treatment was measured in PBS by using dichlorofluorescein substrate. We detected an increase of H_2_O_2_ in all cell lines tested; this induction of H_2_O_2_ was completely blocked by catalase. Addition of 0.5 mM H_2_O_2_ to the cell cultures served as positive control. All H_2_O_2_ measurements were performed in quadruplicates; shown is mean ± SD. (A and B) RPMI: cell culture medium, FCS: foetal calf serum, asc.: ascorbate.

We assessed the spontaneous generation of radicals from ascorbate in medium containing increasing percentage of foetal calf serum (FCS) by using ESR. The addition of ascorbate (8 mM) to the medium yielded a detectable amount of ascorbyl radicals (Asc^−•^; Fig. 1A). Increasing concentrations of FCS (0%, 1%, 10%, 40%, 99%) yielded a correlating increase of detectable Asc^−•^ (Fig. 1A), suggesting that the formation of Asc^−•^ was catalysed by serum components, as previously reported by [3]. At pharmacological concentrations, ascorbate is a precursor of H_2_O_2_ generation in the extracellular milieu [7]. Therefore, the generation of H_2_O_2_ from increasing ascorbate concentrations in nine different cancer cell lines was measured. Addition of ascorbate (8 or 16 mM) resulted in an increase in H_2_O_2_ formation in all of the cancer cell lines (SF268, OVCAR-8, LOX IMVI, K562, NCI-H226, HT29, DU-145, HS-578T, SN12C) independent of the tumour type; as expected, the addition of 100 μg/ml catalase completely destroyed the ascorbate-induced H_2_O_2_ in all nine cell lines (Fig. 1B). Addition of H_2_O_2_ served as positive control (Fig. 1B).

### Ascorbate inhibits proliferation in all 60 cancer cell lines

**Figure 2:**
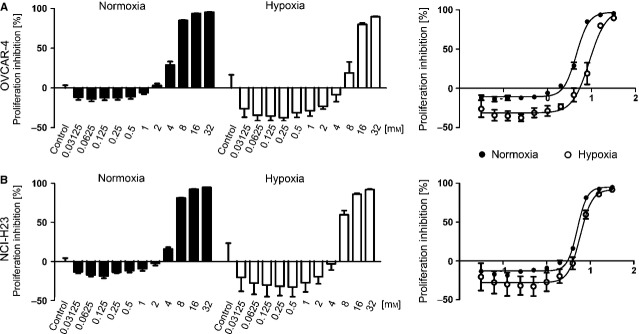
Determination of the IC_50_ concentrations of ascorbate in the OVCAR-4 and NCI-H23 cell lines. Shown are two examples (OVCAR-4 (A), NCI-H23 (B)) of the proliferation assays and the determination of the respective IC_50_ values of ascorbate. The cells were exposed to 11 rising concentrations of ascorbate under normoxic (21% O_2_) and severe hypoxic (0.1% O_2_) conditions. The IC_50_ values were then calculated by using GraphPad Prism 5 (Nonlinear Regression EC_50_ shift; graphs on the right). Hypoxia increased the mean IC_50_ values of OVCAR-4 cells from 4.5 to 9.0 mM, and of NCI-H23 cells from 5.0 to 6.0 mM. Assays were performed in quadruplicates; shown is mean ± SEM.

**Figure 3:**
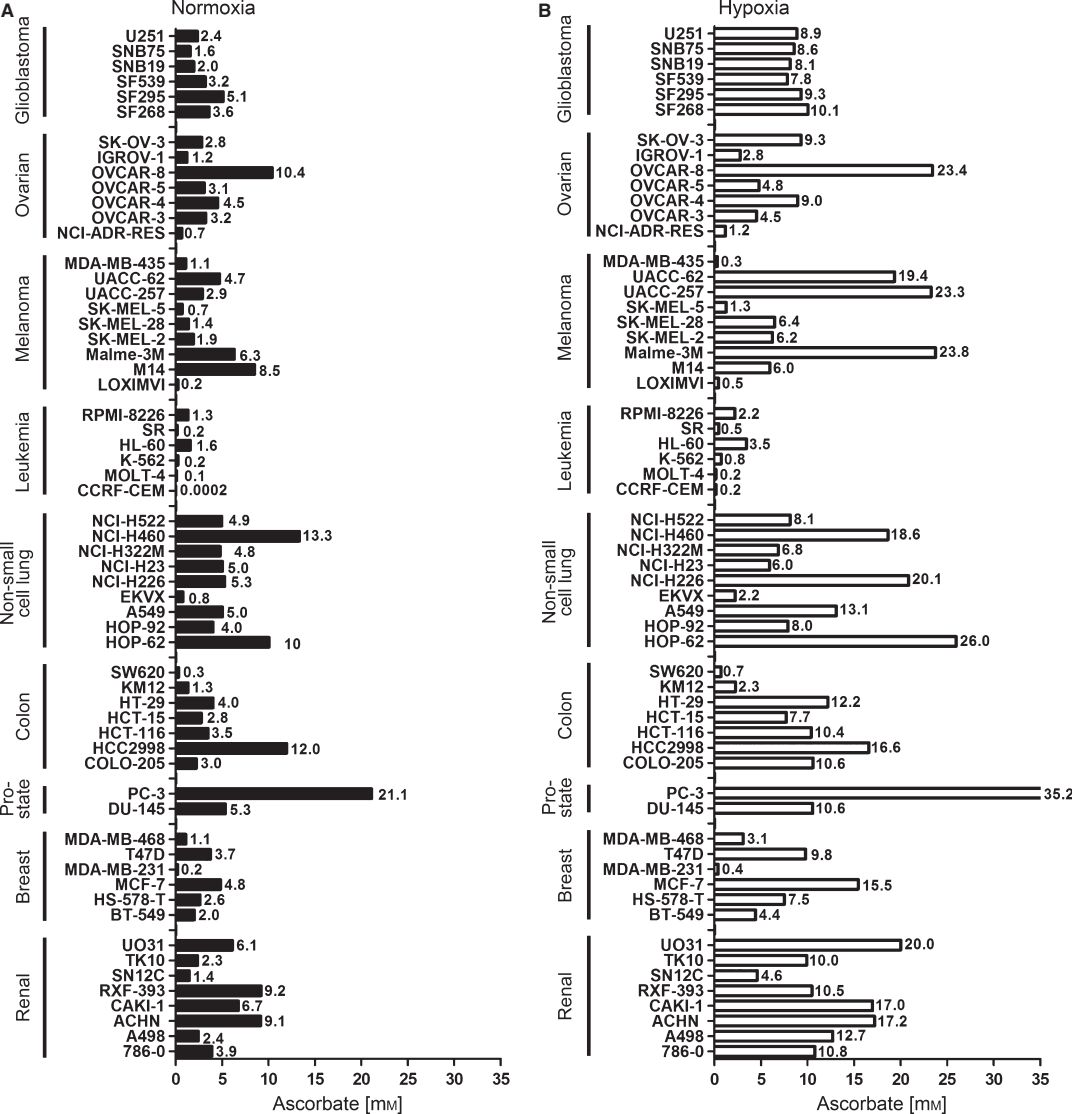
Severe hypoxia significantly increases the IC_50_ concentrations of ascorbate in the 60 cancer cell lines. (A) All 60 cancer cell lines of the NCI60 panel were subjected to the experimental procedures as shown in Figure 2. Shown are the calculated IC_50_ concentrations. (A) IC_50_ concentrations under normoxic conditions. (B) IC_50_ concentrations under hypoxic conditions. Hypoxia increased the mean IC_50_ value of all 60 cancer cell lines from 4.5 ± 3.6 mM to 10.1 ± 5.9 mM (2.2-fold increase, *P* < 0.001, Mann–Whitney *t*-test). Assays were performed in quadruplicates; shown is mean ± SEM.

We next analysed the cytotoxic efficacy of ascorbate on all cell lines of the NCI60 panel. To this end, the cells were incubated with 11 rising concentrations of ascorbate (31.25 μM–32 mM, double concentration in each step) to determine the cytostatic efficacy (inhibition of cell proliferation) for each cell line under normoxic conditions (21% O_2_). The results of two exemplary cell lines (OVCAR-4 and NCI-H23) are displayed in Figure 2. In a second step, the exact IC_50_ concentration was calculated. These two consecutive steps were performed for all 60 cell lines. We found a dose-dependent inhibition of cell proliferation in all cell lines, independent of their mutation status (Table S1) 24 hrs after treatment with pharmacological concentrations achievable in humans *via* i.v. administration of >10 g ascorbate [8] (Fig. 3A). Interestingly, susceptibility towards ascorbate-induced toxicity highly varied from the low micromolar range (leukaemia cell lines) to higher millimolar ranges (*e.g*. lung and prostate cancer cell lines; Fig. 3A). The average IC_50_ value of ascorbate for all 60 cancer cell lines under normoxic conditions was 4.5 ± 3.6 mM. For the different tumour types, we found the following IC_50_ values of ascorbate: renal cancer cell lines (*n* = 8): 5.2 ± 3.1 mM; breast cancer cell lines (*n* = 6): 2.4 ± 1.7 mM; prostate cancer cell lines (*n* = 2): 13.2 ± 11.1 mM; colon cancer cell lines (*n* = 7): 3.7 ± 3.8 mM; lung cancer cell lines (*n* = 9): 5.9 ± 3.6 mM; leukaemia cell lines (*n* = 6): 0.6 ± 0.7 mM; melanoma cell lines (*n* = 9): 3.1 ± 2.8 mM; ovarian cancer cell lines (*n* = 7): 3.7 ± 3.2 mM; glioblastoma cell lines (*n* = 6): 3.0 ± 1.3 mM (Fig. 3A).

### Severe hypoxia significantly increases the IC_50_ concentrations of ascorbate in the 60 cancer cell lines

Simultaneously to the determination of the IC_50_ value for ascorbate under normoxic conditions (21% O_2_), the influence of severe hypoxia (0.1% O_2_) on ascorbate-induced decrease in proliferation was analysed. We chose 0.1% O_2_ treatment for 24 hrs to mimic chronic hypoxia as recently described for prostate cancer cells [23]. To this end, the different cell lines were cultured under hypoxic conditions during ascorbate exposure; hypoxia itself did not induce relevant apoptosis in the cell lines tested. We also detected a dose-dependent inhibition of cell proliferation in all 60 cancer cell lines; however, the IC_50_ value increased for every cell line (mean IC_50_ concentration under normoxia for all cell lines: 4.5 ± 3.6 mM; mean IC_50_ concentration under hypoxia for all cell lines: 10.1 ± 5.9 mM; *P* < 0.001, Mann–Whitney *t*-test; Fig. 3B). This represented a global 2.2-fold increase in ascorbate IC_50_ value. In detail, we found the following IC_50_ values for ascorbate under hypoxic conditions (Fig. 3B; summarized in Table 1): renal cancer: 12.8 ± 5.0 mM, 2.5-fold increase (five of eight cell lines showed a statistically significant increase of the IC_50_ regarding the dose–response curves); breast cancer: 6.8 ± 5.4 mM, 2.8-fold increase (five of six cell lines showed a statistically significant increase of the IC_50_); prostate cancer: 22.9 ± 17.5 mM, 1.7-fold increase (one of two cell lines showed a statistically significant increase of the IC_50_); colon cancer: 8.6 ± 5.6 mM, 2.3-fold increase (four of seven cell lines showed a statistically significant increase of the IC_50_); lung cancer: 12.2 ± 8.0 mM, 2.1-fold increase (four of nine cell lines showed a statistically significant increase of the IC_50_); leukaemia: 1.2 ± 1.3 mM, 2.0-fold increase (five of six cell lines showed a statistically significant increase of the IC_50_); melanoma: 9.7 ± 9.7 mM, 3.1-fold increase (seven of nine cell lines showed a statistically significant increase of the IC_50_); ovarian cancer: 7.9 ± 7.5 mM, 2.1-fold increase (six of seven cell lines showed a statistically significant increase of the IC_50_); glioblastoma: 8.8 ± 0.8 mM, 2.9-fold increase (six of six cell lines showed a statistically significant increase of the IC_50_). The following cell lines did not reach a statistically significant increase of the respective IC_50_ under hypoxia: HT578T, HT-29, HCC2998, HCT-15, CCRF, NCI-H23, NCI-H460, HOP-62, NCI-H322M, NCI-H226, M14, SK-MEL-5, OVCAR-8, PC-3, A498, RXF-393, ACHN.

### Hypoxia-induced ascorbate resistance is driven by HIF1α and by O_2_ partial pressure

**Figure 4:**
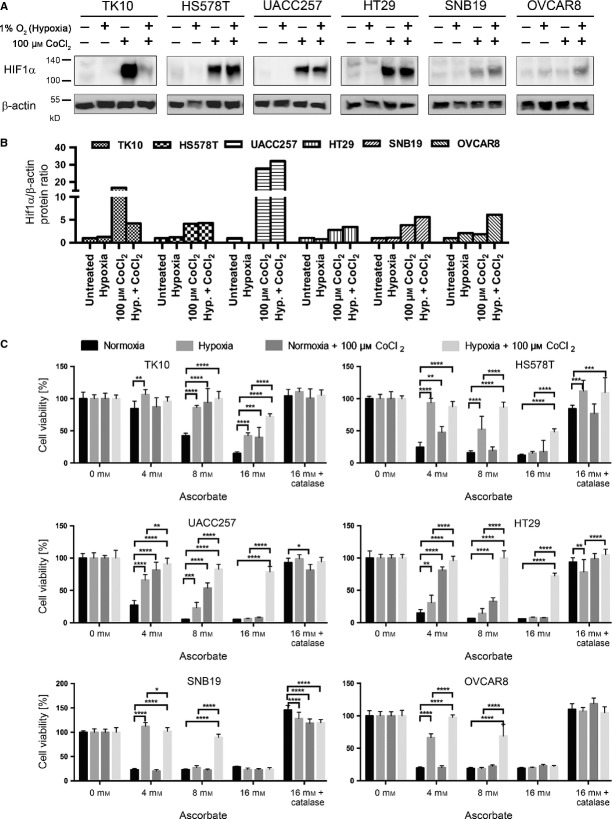
Hypoxia-induced ascorbate resistance is driven by cobalt chloride (CoCl_2_)-induced hypoxia-inducible factor-1α (HIF1α) *and* oxygen pressure. (A and B) 100 μM CoCl_2_ was applied on TK10, HS578T, UACC257, HT29, SNB19 and OVCAR8 cells under normoxic and hypoxic conditions for 24 hrs. The Western blot and densitometric analyses show a strong induction of HIF1α upon CoCl_2_-treatment in five of the six cell lines (C). The influence of hypoxia alone, CoCl_2_-induced HIF1α or both on cancer cell viability after ascorbate exposure at increasing concentrations (0, 4, 8, and 16 mM) for 1 hr was analysed. Hypoxia induced an ascorbate resistance in all of the cells. The same was observed for CoCl_2_-treatment under normoxic conditions in TK10, HS578T, UACC257 and HT29 cells. Their combination (hypoxia + CoCl_2_-treatment) significantly increased the ascorbate resistance in all cells. The addition of catalase blocked the ascorbate-induced proliferation inhibition. N: normoxia (21% O_2_), H: hypoxia (0.1% O_2_). *P < 0.05, **P < 0.01, ***P < 0.001; ****P < 0.0001 (Two-way anova). Assays were performed in sixtuplicates; shown is mean ± SD.

To further determine whether the O_2_-dependent cytotoxic effects of ascorbate were dependent on the hypoxia-inducible factor-1α signalling cascade or simply on O_2_-availability, we applied CoCl_2_, a HIF1α-inducing drug [20] under normoxic and hypoxic conditions to allow the induction of HIF1α. Six cell lines of different tumour entities (TK10, HS578T, UACC257, HT29, SNB19, OVCAR8) that had shown a strong increase in the IC_50_ concentration of ascorbate under hypoxia (refer to Fig. 3B) were incubated with CoCl_2_ for 24 hrs under normoxic or hypoxic conditions. Western blot analyses with subsequent densitometry clearly demonstrated a strong induction of HIF1α upon CoCl_2_-treatment in five of the six cell lines (Fig. 4A and B). Hypoxia alone did not increase the level of detectable HIF1α, probably because of instability of the protein during protein extraction. Next, we analysed the influence of hypoxia alone, HIF1α (induced by CoCl_2_) or both on cancer cell viability after ascorbate exposure at increasing concentrations (0, 4, 8 and 16 mM) for 1 hr. Hypoxia rendered all of the cells resistant towards ascorbate-induced cell death (Fig. 4C). The same was true for CoCl_2_-treatment under normoxic conditions. Interestingly, the combination of both (hypoxia + CoCl_2_-treatment) significantly increased the ascorbate resistance in all of the six tested cancer cells. As expected, the addition of catalase blocked the ascorbate-induced proliferation inhibition (Fig. 4C).

### High-dose ascorbate increases the sub-G1 cell population in the majority of the 60 cancer cell lines

**Figure 5:**
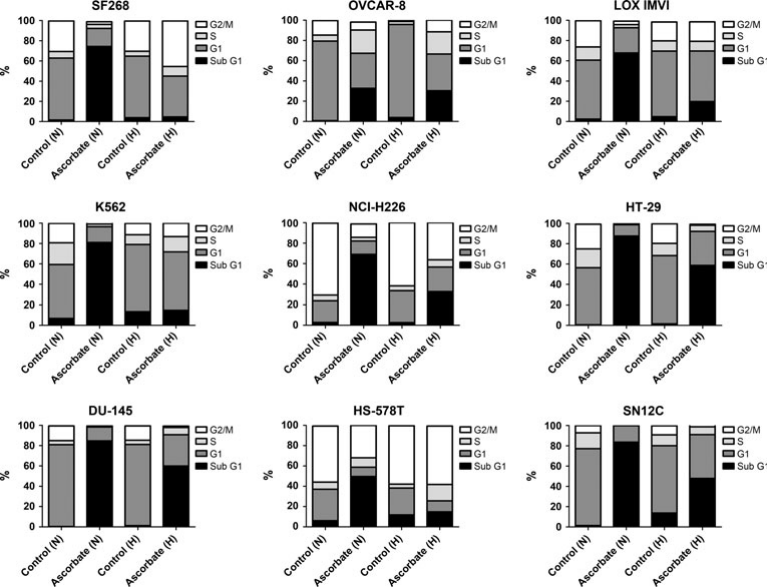
Hypoxia and high-dose ascorbate increase the sub-G1 cell population in the 60 cancer cell lines. FACS cell-cycle analyses (100,000 cells measured per treatment group) were performed on all 60 cancer cell lines after incubation with or without ascorbate at the individual IC_50_ concentration for 24 hrs under normoxic (21% O_2_) and hypoxic (0.1% O_2_) conditions. Depicted is an exemplary cell line of each of the nine tumour entities of the NCI60 panel. The entire data are displayed in Table S2, Ascorbate treatment induced an increase in the sub-G1 cell population (apoptosis) under normoxic and hypoxic conditions. N: normoxia (21% O_2_), H: hypoxia (0.1% O_2_).

To further determine whether the O_2_-dependent cytotoxic effects of ascorbate were conducted by apoptosis induction or *via* alterations in the cell cycle, fluorescence activated cell sorting (FACS) cell-cycle analyses were performed on all 60 cancer cell lines after incubation with ascorbate at the individual IC_50_ concentration for 24 hrs under normoxic (21% O_2_) and hypoxic (0.1% O_2_) conditions. The percentage of cells in the sub-G1 phase (indicating apoptotic cells) after ascorbate incubation varied between the different cell lines (Fig. 5 and Table S2), although the individual IC_50_ concentration of ascorbate determined in the proliferation assay (as described above) was applied in each case. Figure 5 depicts a representative cell line of each of the nine different tumour entities comprised in the NCI60 panel. Details of the cell-cycle distribution under normoxic and hypoxic conditions after ascorbate treatment at the respective IC_50_ concentration of each of the 60 cancer cell lines are given in Table S2. Together, the FACS analyses demonstrate that pharmacological ascorbate primarily induced an increase in the sub-G1 cell population under normoxic conditions in 47 of 60 cells lines (Table S2). Under hypoxic conditions, we observed a comparable result after additional ascorbate treatment: In 45 of 60 cell lines, the sub-G1 cell population was increased. As expected (refer to Fig. 3), the sub-G1 cell population was decreased in the ascorbate + hypoxia group (in 38 of 60 cell lines) compared with the ascorbate + normoxia group.

### Hypoxia and ascorbate treatment alters the expression of GLUT-1 in the 60 cancer cell lines

**Figure 6:**
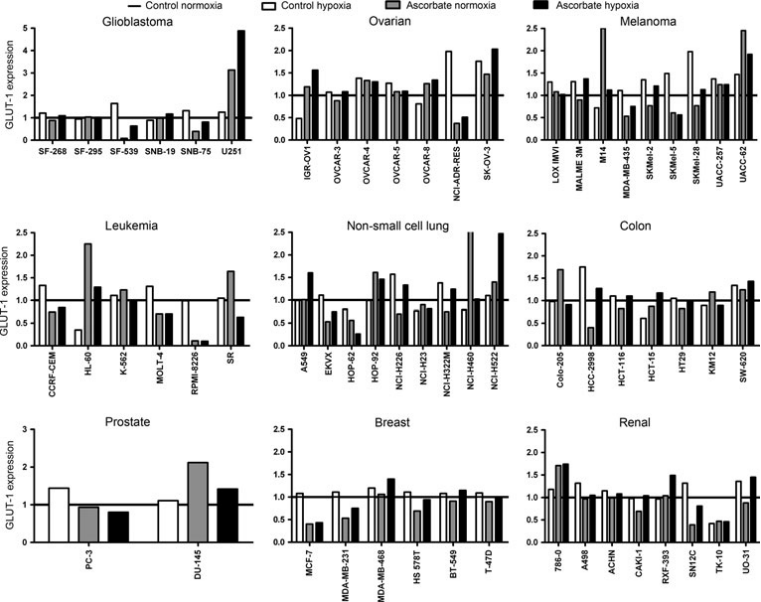
Hypoxia and ascorbate treatment alter the expression of glucose transporter 1 (GLUT-1) in the 60 cancer cell lines. Western blot analyses were performed on the cell lysates of all 60 cell lines 24 hrs after exposure to ascorbate at the individual IC_50_ concentration for 1 hr under normoxic and hypoxic conditions. Protein expression was evaluated by densitometric analyses. Under hypoxia, the GLUT-1 protein level increased in 40 of the cell lines, most pronounced in melanoma and breast cancer cells. Ten cell lines had a reduced GLUT-1 expression under hypoxic conditions (*e.g*. in the non-small cell lung cancer cells). Under normoxia, ascorbate treatment reduced GLUT-1 expression in 30 cell lines, most pronounced in melanoma, non-small cell lung cancer and breast cancer cells; 22 cell lines showed an increased GLUT-1 expression, most pronounced in ovarian and leukaemia cancer cells. Under hypoxia + high-dose ascorbate, 20 cell lines had a reduced GLUT-1 expression, which was most pronounced in the breast cancer cells, while 31 cell lines had an increased GLUT-1 expression, most pronounced in ovarian and colon cancer cells.

Finally, we tested whether the expression of the HIF-1α downstream-target GLUT-1 (responsible for the cellular dehydroascorbate uptake, competitively with glucose, [18]) was regulated by ascorbate in the 60 cell lines. Western blot analyses (and densitometry) were performed on the cell lysates 24 hrs after a 1 hr-exposure to ascorbate at the individual IC_50_ concentration under normoxic and hypoxic conditions. We could not detect a significant correlation between the level of endogenous GLUT-1 expression and susceptibility towards ascorbate-induced cell death (data not shown). However, our results yielded cell type-specific differences in GLUT-1 expression after ascorbate treatment and under hypoxia (Fig. 6). Under hypoxia (0.1% O_2_), the cellular GLUT-1 protein levels increased in 40 of the 60 cell lines. This effect occurred in most of the melanoma and breast cancer cells. This is in line with the fact that GLUT-1 is a downstream-target of HIF-1α, which is induced by hypoxia [24]. However, 10 of the 60 cell lines had a reduced GLUT-1 expression under hypoxic conditions (*e.g*. in the non-small cell lung cancer cell lines). Under normoxic conditions, ascorbate treatment at the individual IC_50_ concentrations decreased GLUT-1 expression in 30 of the 60 cell lines, with the most pronounced effects in melanoma, non-small cell lung cancer and breast cancer cell lines (Fig. 6), while in 22 of the 60 cell lines, an increased GLUT-1 expression was measured (most pronounced effect in ovarian and leukaemia cancer cell lines). In the last experimental setting (high-dose ascorbate + hypoxia), 20 cell lines had a reduced GLUT-1 expression (heterogeneous pattern; most pronounced in the breast cancer cell lines) and 31 cell lines an increased GLUT-1 expression (most pronounced effect in ovarian and colon cancer cell lines; Fig. 6).

## Discussion

The clinical experience from phase I trials shows that in cancer patients, high-dose ascorbate bears only little cytotoxic efficacy [10,19], which is in contrast to *in vitro* and *in vivo* pre-clinical data [3–6]. Cancer cells and the tumour microenvironment are hypoxic [13]. Therefore, in the current study, we asked whether hypoxia might be involved in the reduced efficacy of ascorbate in the killing of cancer cells. As analytical tool, we used the cell lines of the NCI60 panel for our experiments. In summary, our results clearly indicate that the generation of radicals from ascorbate was catalysed by serum and that severe hypoxia (0.1% O_2_) mediated a reduced susceptibility towards ascorbate-induced inhibition of cancer cell proliferation. This ascorbate resistance was enhanced by CoCl_2_-induced HIF1α. However, the ascorbate-induced cytotoxicity did not correlate with the endogenous GLUT-1 expression.

In a first approach, the individual IC_50_ values for ascorbate were determined for all 60 cell lines under normoxic (21% O_2_) and hypoxic (0.1% O_2_) conditions. Under normoxic conditions, all cell lines had IC_50_ values that were in the range of concentrations that can be achieved in patients by i.v. administration of ascorbate [4]. Hypoxia significantly increased the IC_50_ values. This might account for the fact that in pilot clinical trials on stage IV cancer patients by using high-dose pharmacological ascorbate, the cytotoxic efficacy was lower [10] than in defined cell culture experiments conducted under normoxic conditions [3]. Secondly, we asked whether the reduced susceptibility of the cancer cells towards ascorbate under hypoxic conditions was merely driven by the reduced O_2_ partial pressure (essential for radical formation) or whether HIF1α-signalling might also be involved in the observed cellular resistance. We used CoCl_2_ as inductor for HIF1α [22]. Our data show that both the decreased O_2_ partial pressure (hypoxia) *and* the expression of HIF1α drove resistance towards ascorbate-induced cell death. To determine the mode of action of ascorbate-induced inhibition of cancer cell proliferation in more detail, we performed FACS cell-cycle analyses on all 60 cell lines after incubation with ascorbate at the individual IC_50_ under normoxic and hypoxic conditions. The FACS analyses yielded an increased sub-G1 (apoptotic) population in the majority of the cell lines after incubation with ascorbate under normoxic conditions, which was significantly reduced under hypoxic conditions. The high percentage of cells in the sub-G1 phase after incubation with ascorbate indicated that the induction of apoptosis was the major antiproliferative mode of action of ascorbate in cancer cells. However, in some of the cell lines analysed, no apoptosis induction was observed despite the obvious growth inhibition after incubation at the respective IC_50_ concentration. In such cases, pyknosis/necrosis probably caused the cytotoxic effects [3]. Finally, we analysed a possible role for GLUT-1 (which acts as pro-survival molecule in cancer cells [25]) in the susceptibility towards ascorbate-induce cell death. Western blot analyses demonstrated that hypoxic conditions increased the expression of the GLUT-1 protein in the majority of the 60 cell lines. Interestingly, ascorbate treatment decreased GLUT-1 expression under normoxic conditions in 30 of the cell lines. Moreover, under hypoxic conditions, ascorbate treatment reduced the pro-survival capacity for GLUT-1 up-regulation in nine of the 40 cell lines (from 40 to 31) and doubled the number of cell lines with a decreased expression of GLUT-1 under hypoxia alone (from 10 to 20), suggesting that high-dose ascorbate interferes with pro-survival mechanisms in the cancer cells. A limitation of the Western blot results on GLUT-1 expression is the impossibility of statistical quantification of the protein expression, as the Western blot experiments were only conducted once for each of the 60 cell lines at the four different experimental conditions, thus only allowing a qualitative analysis. As oxygenation (HBO treatment) of cancer patients does not increase tumour growth or the recurrence rate [16], our data imply that for future clinical application, cancer patients should be oxygenized before and during ascorbate infusions to maximize the cytotoxic efficacy.

### Conclusion

Our results demonstrate that pharmacological doses of ascorbate bear cytotoxic effects on the NCI60 panel of cancer cells *in vitro*. This cytotoxicity is executed by ascorbyl radicals and H_2_O_2_ and is catalysed by serum components. Hypoxic conditions and HIF-1α-signalling, both present in cancer metastases, confer resistance to the cancer cells towards ascorbate-induced cytotoxicity, possibly *via* up-regulation of pro-survival HIF-1α downstream-targets (*e.g*. GLUT-1). At the same time, under hypoxic conditions, pharmacological ascorbate treatment partly reduces the expression of GLUT-1. These findings might be the key for the lack of clinical efficacy of high-dose ascorbate in cancer patients up to this day. We conclude that in future clinical trials, the respective patients should be oxygenized in addition to ascorbate therapy to break drug resistance.

## Supporting information

Table S1 Source of the 60 cancer cell lines, and cell line-specific mutations.

Table S2 Cell-cycle FACS analyses of the 60 cancer cell lines und normoxia and hypoxia, with and without ascorbate treatment at the individual IC_50_ concentration.
