# Supplementary material for: The ROS-induced cytotoxicity of ascorbate is attenuated by hypoxia and HIF-1alpha in the NCI60 cancer cell lines
Source: J Cell Mol Med. 2013 Dec 14;18(3):530–41. doi: 10.1111/jcmm.12207 (PMC3955158; doi:10.1111/jcmm.12207)
Supplement: Table S1 — Source of the 60 cancer cell lines, and cell line-specific mutations. [file jcmm0018-0530-sd1.doc]

Table S1: Source of the 60 cancer cell lines, and cell line-specific mutations
