# Supplementary material for: The ROS-induced cytotoxicity of ascorbate is attenuated by hypoxia and HIF-1alpha in the NCI60 cancer cell lines
Source: J Cell Mol Med. 2013 Dec 14;18(3):530–41. doi: 10.1111/jcmm.12207 (PMC3955158; doi:10.1111/jcmm.12207)
Supplement: Table S2 — Cell-cycle FACS analyses of the 60 cancer cell lines und normoxia and hypoxia, with and without ascorbate treatment at the individual IC50 concentration. [file jcmm0018-0530-sd2.doc]

| **Cell line** | **Treatment** | **sub G1 [%]** | **G1 [%]** | **S [%]** | **G2/M [%]** |
| --- | --- | --- | --- | --- | --- |
| **Glioblastoma** | | | | | |
| U251 | control (N) | 4 | 16,5 | 9 | 70,6 |
| U251 | ascorbate (N) | 85,7 | 9,5 | 3,4 | 0,9 |
| U251 | control (H) | 1,6 | 45,1 | 17,4 | 34,9 |
| U251 | ascorbate (H) | 7,6 | 57 | 22 | 11 |
| SNB75 | control (N) | 15,5 | 55,5 | 3,6 | 25,2 |
| SNB75 | ascorbate (N) | 89,3 | 9,1 | 0,9 | 0,7 |
| SNB75 | control (H) | 33,6 | 43,7 | 3,5 | 19,1 |
| SNB75 | ascorbate (H) | 100 | 0 | 0 | 0 |
| SNB19 | control (N) | 0,5 | 75,4 | 5,4 | 18,3 |
| SNB19 | ascorbate (N) | 69,2 | 24,5 | 2,7 | 2,9 |
| SNB19 | control (H) | 0,3 | 74 | 5,9 | 19,7 |
| SNB19 | ascorbate (H) | 48,8 | 41,9 | 4,4 | 2,6 |
| SF539 | control (N) | 2,8 | 59,8 | 13,6 | 20,3 |
| SF539 | ascorbate (N) | 7,7 | 5,4 | 23,1 | 58,4 |
| SF539 | control (H) | 4,4 | 67,1 | 6 | 12,3 |
| SF539 | ascorbate (H) | 47,5 | 5 | 4,3 | 26 |
| SF295 | control (N) | 3,9 | 59,7 | 8,3 | 24,7 |
| SF295 | ascorbate (N) | 28,3 | 4,2 | 6,7 | 60,3 |
| SF295 | control (H) | 4,8 | 64,6 | 13,1 | 25,6 |
| SF295 | ascorbate (H) | 3,4 | 9,1 | 30,6 | 65,3 |
| SF268 | control (N) | 1,5 | 61,4 | 6,5 | 30,2 |
| SF268 | ascorbate (N) | 74,4 | 17,8 | 4 | 2,6 |
| SF268 | control (H) | 3,9 | 60,9 | 4,8 | 29,9 |
| SF268 | ascorbate (H) | 4,6 | 40,4 | 9,5 | 45,1 |
| **Ovarian cancer** | | | | | |
| SK-OV-3 | control (N) | 4,5 | 82,4 | 2,9 | 10,1 |
| SK-OV-3 | ascorbate (N) | 81,7 | 17 | 0,5 | 0,4 |
| SK-OV-3 | control (H) | 4,4 | 83,8 | 3,2 | 8,5 |
| SK-OV-3 | ascorbate (H) | 2,9 | 4,5 | 29,2 | 63,3 |
| IGROV-1 | control (N) | 11,4 | 61,6 | 4,6 | 22,2 |
| IGROV-1 | ascorbate (N) | 87,1 | 11,2 | 0,6 | 1,1 |
| IGROV-1 | control (H) | 19,7 | 42,1 | 2,7 | 35,4 |
| IGROV-1 | ascorbate (H) | 89,3 | 9,7 | 0 | 1,1 |
| OVCAR-8 | control (N) | 0,8 | 78,6 | 5,9 | 14,3 |
| OVCAR-8 | ascorbate (N) | 32,7 | 34,7 | 22,9 | 7,8 |
| OVCAR-8 | control (H) | 3,9 | 91,9 | 3 | 1,2 |
| OVCAR-8 | ascorbate (H) | 30,3 | 36,3 | 21,9 | 11,5 |
| OVCAR-5 | control (N) | 0,2 | 83,4 | 3,3 | 12,8 |
| OVCAR-5 | ascorbate (N) | 88,2 | 10,7 | 0,6 | 0,5 |
| OVCAR-5 | control (H) | 2,6 | 81 | 3,2 | 13,3 |
| OVCAR-5 | ascorbate (H) | 73,4 | 23,2 | 2,5 | 1 |
| OVCAR-4 | control (N) | 4,9 | 54,6 | 15,1 | 23,7 |
| OVCAR-4 | ascorbate (N) | 6,3 | 9,2 | 29,7 | 52,8 |
| OVCAR-4 | control (H) | 3,7 | 57,8 | 15,1 | 21,9 |
| OVCAR-4 | ascorbate (H) | 4,5 | 39,6 | 15,7 | 38,8 |
| OVCAR-3 | control (N) | 12,5 | 47,2 | 3,7 | 36,2 |
| OVCAR-3 | ascorbate (N) | 98,2 | 1,6 | 0,1 | 0,1 |
| OVCAR-3 | control (H) | 29,3 | 32,8 | 2,6 | 35 |
| OVCAR-3 | ascorbate (H) | 87,1 | 9,3 | 0,6 | 2,5 |
| NCI-ADR-RES | control (N) | 0,5 | 55,9 | 12,4 | 30,9 |
| NCI-ADR-RES | ascorbate (N) | 0,7 | 0,7 | 8 | 90,6 |
| NCI-ADR-RES | control (H) | 4,1 | 75 | 9,4 | 10,6 |
| NCI-ADR-RES | ascorbate (H) | 1,9 | 0,8 | 0,3 | 97 |
| **Melanoma** | | | | | |
| MDA MB 435 | control (N) | 1,8 | 62,6 | 6,4 | 28,8 |
| MDA MB 435 | ascorbate (N) | 0,9 | 22,8 | 28,3 | 47,1 |
| MDA MB 435 | control (H) | 4,3 | 27,3 | 7 | 61,2 |
| MDA MB 435 | ascorbate (H) | 43,4 | 34 | 7,5 | 14,6 |
| UACC-62 | control (N) | 2,8 | 80 | 5,2 | 11,8 |
| UACC-62 | ascorbate (N) | 5 | 42,9 | 10,7 | 40,1 |
| UACC-62 | control (H) | 3,8 | 82,3 | 4,8 | 8,9 |
| UACC-62 | ascorbate (H) | 13,5 | 51,1 | 19,7 | 15,4 |
| UACC-257 | control (N) | 34,3 | 52,6 | 2,3 | 10,6 |
| UACC-257 | ascorbate (N) | 34,6 | 43,2 | 7,8 | 12,7 |
| UACC-257 | control (H) | 29,1 | 57,4 | 3,6 | 9,4 |
| UACC-257 | ascorbate (H) | 14 | 47,1 | 6 | 32,2 |
| SKMel5 | control (N) | 3,6 | 71,3 | 11,7 | 12,1 |
| SKMel5 | ascorbate (N) | 99,8 | 0,1 | 0 | 0,1 |
| SKMel5 | control (H) | 9,9 | 72,8 | 6,9 | 10,1 |
| SKMel5 | ascorbate (H) | 98,3 | 1,5 | 0 | 0,1 |
| SKMel28 | control (N) | 2 | 63,3 | 20,1 | 13,3 |
| SKMel28 | ascorbate (N) | 3,7 | 30 | 26,7 | 37,6 |
| SKMel28 | control (H) | 1,8 | 86,8 | 4 | 7,2 |
| SKMel28 | ascorbate (H) | 0,7 | 69,9 | 18,2 | 10,7 |
| SKMel2 | control (N) | 5 | 55,4 | 9,2 | 30,4 |
| SKMel2 | ascorbate (N) | 62,3 | 23,8 | 9,5 | 3,3 |
| SKMel2 | control (H) | 6,1 | 51,2 | 11,8 | 31,4 |
| SKMel2 | ascorbate (H) | 6 | 5,7 | 24,8 | 63,6 |
| MALME 3M | control (N) | 0,6 | 73,6 | 8,2 | 17,1 |
| MALME 3M | ascorbate (N) | 3,5 | 62,2 | 9,4 | 24,1 |
| MALME 3M | control (H) | 1,9 | 78,6 | 5 | 14,2 |
| MALME 3M | ascorbate (H) | 1,2 | 69,2 | 7,3 | 22 |
| M14 | control (N) | 0,4 | 71 | 9,7 | 18,7 |
| M14 | ascorbate (N) | 1 | 25,7 | 18 | 54,4 |
| M14 | control (H) | 1,4 | 74,7 | 5,5 | 18,1 |
| M14 | ascorbate (H) | 45,7 | 44,5 | 5,9 | 2,4 |
| LOX IMVI | control (N) | 2,4 | 58,3 | 13 | 25,9 |
| LOX IMVI | ascorbate (N) | 67,8 | 24,9 | 3,1 | 3,4 |
| LOX IMVI | control (H) | 4,8 | 64,8 | 10,1 | 18,7 |
| LOX IMVI | ascorbate (H) | 19,7 | 50 | 9,6 | 19,2 |
| **Leukemia** | | | | | |
| RPMI 8226 | control (N) | 48,5 | 22,2 | 6 | 19,5 |
| RPMI 8226 | ascorbate (N) | 84,5 | 6,8 | 0,7 | 0,4 |
| RPMI 8226 | control (H) | 54,7 | 15,6 | 6,1 | 20,8 |
| RPMI 8226 | ascorbate (H) | 75,3 | 13,4 | 4,7 | 3,9 |
| SR | control (N) | 29,4 | 48,1 | 6,9 | 15 |
| SR | ascorbate (N) | 64,6 | 23,8 | 7,2 | 4,3 |
| SR | control (H) | 43,1 | 46,8 | 2,9 | 7,2 |
| SR | ascorbate (H) | 60,8 | 30,2 | 4,9 | 4,1 |
| HL-60 | control (N) | 10,4 | 52,9 | 17,1 | 15,9 |
| HL-60 | ascorbate (N) | 95,8 | 2,6 | 0,4 | 0,2 |
| HL-60 | control (H) | 21,6 | 49,6 | 19,1 | 15,7 |
| HL-60 | ascorbate (H) | 82,6 | 14,9 | 2,7 | 1,7 |
| K562 | control (N) | 6,8 | 52,6 | 21,3 | 19,1 |
| K562 | ascorbate (N) | 81 | 15,5 | 2,6 | 1 |
| K562 | control (H) | 13,4 | 65,6 | 9,6 | 11,2 |
| K562 | ascorbate (H) | 14,7 | 57,1 | 15 | 13,2 |
| MOLT-4 | control (N) | 1,8 | 54,3 | 12,9 | 30,7 |
| MOLT-4 | ascorbate (N) | 97,2 | 2,2 | 0,4 | 0,2 |
| MOLT-4 | control (H) | 49 | 3,7 | 4,1 | 43 |
| MOLT-4 | ascorbate (H) | 37,4 | 45,7 | 11,1 | 5,3 |
| CCRF CEM | control (N) | 1,5 | 42 | 31,1 | 25 |
| CCRF CEM | ascorbate (N) | 91,3 | 4,9 | 3,1 | 0,4 |
| CCRF CEM | control (H) | 1,6 | 49,2 | 27,1 | 21,5 |
| CCRF CEM | ascorbate (H) | 100 | 0 | 0 | 0 |
| **Non-small cell lung cancer** | | | | | |
| NCI-522 | control (N) | 2 | 67,4 | 9,2 | 21,2 |
| NCI-522 | ascorbate (N) | 7,5 | 54,1 | 12,9 | 25,8 |
| NCI-522 | control (H) | 4,5 | 77,5 | 5 | 13 |
| NCI-522 | ascorbate (H) | 5,4 | 57,7 | 11,7 | 24,9 |
| NCI-H460 | control (N) | 2 | 69,4 | 10,9 | 17 |
| NCI-H460 | ascorbate (N) | 12,9 | 12 | 27,5 | 46 |
| NCI-H460 | control (H) | 2,6 | 72,6 | 6,6 | 17,7 |
| NCI-H460 | ascorbate (H) | 99,9 | 0,1 | 0 | 0 |
| NCI-H322M | control (N) | 2,4 | 47,9 | 9,5 | 39,9 |
| NCI-H322M | ascorbate (N) | 22,4 | 28,5 | 10,5 | 37,8 |
| NCI-H322M | control (H) | 0,9 | 48,4 | 8,8 | 42,8 |
| NCI-H322M | ascorbate (H) | 19,5 | 22 | 10,7 | 46,5 |
| NCI-H23 | control (N) | 0,7 | 58,9 | 14 | 25,7 |
| NCI-H23 | ascorbate (N) | 1,6 | 28,3 | 32,4 | 35,9 |
| NCI-H23 | control (H) | 2 | 67,5 | 8,6 | 21,6 |
| NCI-H23 | ascorbate (H) | 1,4 | 30,1 | 25,7 | 41,7 |
| NCI-H226 | control (N) | 2,8 | 20,7 | 5,6 | 70,5 |
| NCI-H226 | ascorbate (N) | 69 | 13,1 | 3,6 | 13,1 |
| NCI-H226 | control (H) | 2,6 | 30,9 | 4,7 | 61,8 |
| NCI-H226 | ascorbate (H) | 32,5 | 24,1 | 7,1 | 36,3 |
| EKVX | control (N) | 0,9 | 68,9 | 5,5 | 24,4 |
| EKVX | ascorbate (N) | 95,1 | 3,8 | 0,5 | 0,5 |
| EKVX | control (H) | 1 | 82,8 | 6,4 | 9,7 |
| EKVX | ascorbate (H) | 60,6 | 33,5 | 2,6 | 1,9 |
| A549 | control (N) | 1,4 | 51,5 | 22 | 24,7 |
| A549 | ascorbate (N) | 7,3 | 1 | 1,7 | 90 |
| A549 | control (H) | 5,3 | 64,2 | 14,8 | 15,2 |
| A549 | ascorbate (H) | 27,8 | 9,2 | 5,9 | 56,9 |
| HOP-92 | control (N) | 4,1 | 57,7 | 10,9 | 27 |
| HOP-92 | ascorbate (N) | 33,9 | 18,6 | 19,4 | 27,6 |
| HOP-92 | control (H) | 2,3 | 61,2 | 8,8 | 27,1 |
| HOP-92 | ascorbate (H) | 49,1 | 20,3 | 13,5 | 16,6 |
| HOP-62 | control (N) | 18,4 | 63,9 | 7,4 | 9,1 |
| HOP-62 | ascorbate (N) | 4,7 | 28 | 19 | 46,8 |
| HOP-62 | control (H) | 14,2 | 64,8 | 9,2 | 11,6 |
| HOP-62 | ascorbate (H) | 4,8 | 30 | 15,8 | 49,3 |
| **Colon cancer** | | | | | |
| SW620 | control (N) | 1,3 | 56 | 18,3 | 26,8 |
| SW620 | ascorbate (N) | 86,6 | 10,1 | 1,5 | 0,8 |
| SW620 | control (H) | 0,8 | 66,9 | 16,3 | 22,6 |
| SW620 | ascorbate (H) | 56,3 | 40 | 2,5 | 2,1 |
| KM12 | control (N) | 4,3 | 53,3 | 14,5 | 24,7 |
| KM12 | ascorbate (N) | 4,3 | 53,3 | 14,5 | 24,7 |
| KM12 | control (H) | 4 | 52 | 13,9 | 29,2 |
| KM12 | ascorbate (H) | 94,3 | 4,3 | 0,9 | 0,4 |
| HT-29 | control (N) | 0,6 | 55,8 | 18,5 | 24,3 |
| HT-29 | ascorbate (N) | 87,5 | 11,2 | 1 | 0,3 |
| HT-29 | control (H) | 1,5 | 66,8 | 12 | 19,3 |
| HT-29 | ascorbate (H) | 58,6 | 33,5 | 6 | 1 |
| HCT-15 | control (N) | 2,5 | 62,4 | 7,4 | 26,2 |
| HCT-15 | ascorbate (N) | 90,6 | 7,5 | 0,6 | 1,3 |
| HCT-15 | control (H) | 10,7 | 47,4 | 9,4 | 30,2 |
| HCT-15 | ascorbate (H) | 6,5 | 42,7 | 10,9 | 37,4 |
| HCT-116 | control (N) | 1,1 | 86,3 | 2,7 | 9,5 |
| HCT-116 | ascorbate (N) | 29,1 | 27,5 | 24,6 | 17,7 |
| HCT-116 | control (H) | 1,7 | 69,4 | 5,9 | 22,9 |
| HCT-116 | ascorbate (H) | 82,8 | 15,3 | 1,2 | 0,6 |
| HCC 2998 | control (N) | 12,9 | 45,4 | 16,1 | 24,9 |
| HCC 2998 | ascorbate (N) | 13,4 | 6,9 | 8,2 | 71,1 |
| HCC 2998 | control (H) | 16,8 | 50,2 | 11,2 | 20,9 |
| HCC 2998 | ascorbate (H) | 31,8 | 10,2 | 7 | 50,6 |
| COLO 205 | control (N) | 1,5 | 66,6 | 9,1 | 22,2 |
| COLO 205 | ascorbate (N) | 1,6 | 24 | 21,4 | 52,2 |
| COLO 205 | control (H) | 4,3 | 51,9 | 14,7 | 27,4 |
| COLO 205 | ascorbate (H) | 73,1 | 18,9 | 4 | 3,3 |
| **Prostate cancer** | | | | | |
| PC-3 | control (N) | 5 | 77,9 | 6,2 | 10,5 |
| PC-3 | ascorbate (N) | 80,7 | 17,9 | 1,2 | 0,2 |
| PC-3 | control (H) | 2,1 | 58,9 | 13,2 | 25,1 |
| PC-3 | ascorbate (H) | 38,8 | 39,5 | 15,9 | 5,1 |
| DU-145 | control (N) | 0,3 | 80,6 | 4 | 14,5 |
| DU-145 | ascorbate (N) | 84,6 | 13,7 | 0,7 | 0,2 |
| DU-145 | control (H) | 1,1 | 80 | 4,2 | 14,1 |
| DU-145 | ascorbate (H) | 59,9 | 30,8 | 7,1 | 1,3 |
| **Breast cancer** | | | | | |
| MDA MB 468 | control (N) | 1,1 | 66,9 | 8,4 | 23,3 |
| MDA MB 468 | ascorbate (N) | 69,6 | 19,7 | 5,4 | 5 |
| MDA MB 468 | control (H) | 1,9 | 24,6 | 28,7 | 44,3 |
| MDA MB 468 | ascorbate (H) | 60,1 | 28,6 | 9,1 | 1,9 |
| T47D | control (N) | 1,7 | 71,2 | 6,6 | 20,4 |
| T47D | ascorbate (N) | 1,9 | 58,8 | 16,1 | 21,8 |
| T47D | control (H) | 1,5 | 73,4 | 9,3 | 15,2 |
| T47D | ascorbate (H) | 3,9 | 56,2 | 17,6 | 20,5 |
| MDA MB 231 | control (N) | 0,3 | 57,6 | 13,5 | 27,8 |
| MDA MB 231 | ascorbate (N) | 0,4 | 8,4 | 22,8 | 67,2 |
| MDA MB 231 | control (H) | 5,2 | 61,8 | 14,5 | 18 |
| MDA MB 231 | ascorbate (H) | 0,5 | 21 | 32,3 | 45,6 |
| MCF-7 | control (N) | 1 | 61,5 | 12,4 | 24,6 |
| MCF-7 | ascorbate (N) | 43,9 | 14 | 25,2 | 16,8 |
| MCF-7 | control (H) | 1,1 | 63,1 | 9,9 | 25,3 |
| MCF-7 | ascorbate (H) | 7,1 | 39,6 | 19,9 | 31 |
| HS-578T | control (N) | 6 | 31,1 | 7 | 55,2 |
| HS-578T | ascorbate (N) | 49,6 | 9,2 | 9,2 | 31,9 |
| HS-578T | control (H) | 11,8 | 26,5 | 3,8 | 57,4 |
| HS-578T | ascorbate (H) | 14,8 | 10,8 | 16,2 | 57,5 |
| BT 549 | control (N) | 47,5 | 34 | 8,8 | 8,9 |
| BT 549 | ascorbate (N) | 71,5 | 23 | 2,5 | 1 |
| BT 549 | control (H) | 33,8 | 54,6 | 6,9 | 3,7 |
| BT 549 | ascorbate (H) | 27 | 69,4 | 1,6 | 1,1 |
| **Renal cancer** | | | | | |
| UO31 | control (N) | 1 | 54,6 | 5,8 | 38,6 |
| UO31 | ascorbate (N) | 4,7 | 4,8 | 13,5 | 77 |
| UO31 | control (H) | 0,9 | 55,8 | 2,8 | 40,4 |
| UO31 | ascorbate (H) | 14,5 | 5,9 | 15,1 | 64,6 |
| TK10 | control (N) | 2,5 | 70,1 | 8,3 | 18,9 |
| TK10 | ascorbate (N) | 53,3 | 3,6 | 2,4 | 40 |
| TK10 | control (H) | 2,9 | 84,7 | 3,2 | 8,7 |
| TK10 | ascorbate (H) | 50 | 5,6 | 8,3 | 36,1 |
| SNC12C | control (N) | 1,4 | 75,8 | 15,6 | 7,1 |
| SNC12C | ascorbate (N) | 83,6 | 16,2 | 0,2 | 0 |
| SNC12C | control (H) | 13,7 | 66,4 | 10,7 | 8,9 |
| SNC12C | ascorbate (H) | 47,9 | 43,1 | 7,7 | 1,8 |
| RXF-393 | control (N) | 11,9 | 27,7 | 10,8 | 38,7 |
| RXF-393 | ascorbate (N) | 17,1 | 0,3 | 0,3 | 71 |
| RXF-393 | control (H) | 31,8 | 21,6 | 6,9 | 26,9 |
| RXF-393 | ascorbate (H) | 14,7 | 23,2 | 9,1 | 44,3 |
| CAKI-1 | control (N) | 2,8 | 85,7 | 2,3 | 8,8 |
| CAKI-1 | ascorbate (N) | 3,8 | 44,6 | 19,6 | 30,4 |
| CAKI-1 | control (H) | 0,8 | 86 | 1,8 | 10,8 |
| CAKI-1 | ascorbate (H) | 2 | 50,5 | 12,4 | 32,3 |
| ACHN | control (N) | 7,1 | 79,1 | 2,9 | 10,9 |
| ACHN | ascorbate (N) | 86,7 | 11,4 | 0,8 | 0,4 |
| ACHN | control (H) | 18,9 | 58,6 | 1,9 | 20,6 |
| ACHN | ascorbate (H) | 93 | 5,6 | 0,4 | 0,9 |
| A498 | control (N) | 88,8 | 10,6 | 0,1 | 0 |
| A498 | ascorbate (N) | 10,8 | 75,2 | 3,1 | 10,7 |
| A498 | control (H) | 13,7 | 19,1 | 19,5 | 47,5 |
| A498 | ascorbate (H) | 14,2 | 71,7 | 3,5 | 10 |
| 786-0 | control (N) | 1,9 | 74,6 | 7,1 | 16,3 |
| 786-0 | ascorbate (N) | 33,2 | 37,1 | 20,6 | 8,8 |
| 786-0 | control (H) | 1,8 | 71,2 | 7,3 | 19,4 |
| 786-0 | ascorbate (H) | 5,7 | 55 | 12,1 | 26,9 |
|  |  |  |  |  |  |
| N: normoxia |  |  |  |  |  |
| H: hypoxia |  |  |  |  |  |
|  |  |  |  |  |  |
| **Table S2**: Cell-cycle FACS-analyses of the 60 cancer cell lines und normoxia and hypoxia, with and without ascorbate treatment at the individual IC_50_ concentration. | | | | | |

**Red: subG1 (ascorbate N) > subG1 (control N)**

**Orange: subG1 (ascorbate H) > subG1 (control H)**

**Green: subG1 (ascorbate N) > subG1 (ascorbate H)**

**Blue: G2/M (ascorbate H) > G2/M (control H)**
